# A high risk of sleep apnea is associated with less postoperative cognitive dysfunction after intravenous anesthesia: results of an observational pilot study

**DOI:** 10.1186/s12871-018-0602-9

**Published:** 2018-10-02

**Authors:** Soeren Wagner, Joerg Quente, Sven Staedtler, Katharina Koch, Tanja Richter-Schmidinger, Johannes Kornhuber, Harald Ihmsen, Juergen Schuettler

**Affiliations:** 1 Department of Anesthesiology, University Hospital Erlangen, Friedrich-Alexander-University Erlangen-Nuremberg, Erlangen, Germany; 2 Department of Anesthesiology, Katharinenhospital Klinikum Stuttgart, Kriegsbergstrasse 60, D-70174 Stuttgart, Germany; 3 Department of Psychiatry and Psychotherapy, University Hospital Erlangen, Friedrich-Alexander-University, Erlangen, Germany; 4 Department of Anaesthesiology, Klinikum Oldenburg AöR, University Hospital Oldenburg, Oldenburg, Germany

**Keywords:** Cognitive dysfunction, Sleep apnea, Intravenous anesthesia, Preconditioning

## Abstract

**Background:**

The obstructive sleep apnea syndrome (OSAS) is characterized by temporary cerebral hypoxia which can cause cognitive dysfunction. On the other hand, hypoxia induced neurocognitive deficits are detectable after general anesthesia.

The objective of this study was to evaluate the impact of a high risk of OSAS on the postoperative cognitive dysfunction after intravenous anesthesia.

**Methods:**

In this single center trial between June 2012 and June 2013 43 patients aged 55 to 80 years with an estimated hospital stay of at least 3 days undergoing surgery were enrolled. Patients were screened for a high risk of OSAS using the STOP-BANG test. The cognitive function was assessed using a neuropsychological test battery, including the DemTect test for cognitive impairment and the RMBT test for memory, the day before surgery and within 36 h after extubation.

**Results:**

Twenty-two of the 43 analyzed patients were identified as patients with a high risk of OSAS. Preoperatively, OSAS patients showed a significant worse performance only for the DemTect (*p* = 0.0043). However, when comparing pre- and postoperative test results, the OSAS patients did not show a significant loss in any test but significantly improved in RMBT test, whereas the control group showed a significant worse performance in three of eight tests. In five tests, we found a significant difference between the two groups with respect to the change from pre- to postoperative cognitive function.

**Conclusion:**

Patients with a high risk of OSAS showed a less impairment of memory function and work memory performance after intravenous anesthesia. This might be explained by a beneficial effect of intrinsic hypoxic preconditioning in these patients.

## Background

The Obstructive Sleep Apnea Syndrome (OSAS) is the most common type of sleep apnea disorder. It is caused by an obstruction of the upper airway and causes dramatic arterial oxygen desaturation during sleep. The disease is characterized by repetitive episodes of hypopnea or apnea with intermittent arousals and considerable disruption of normal sleep architecture [1]. Epidemiological findings suggest that merely 2% of middle-aged (30–60 years of age) women and 4% of middle-aged (30–60 years of age) men are affected, however almost 75% of OSAS patients still remain undiagnosed [2]. OSAS patients are seriously affected by daytime sleepiness, fatigue and neuropsychological impairment compromising several areas of cognitive function including attention, vigilance, memory and executive function. Harmful hypoxemic intervals are also frequently observed in the immediate phase after general anesthesia procedures. The incidence of hypoxic episodes in the post anesthesia unit has been reported to be between 7.6% [3] and 13.5% [4]. These repeated episodes of desaturation often occur unrecognized and prolonged [5]. On the other hand, there is clinical evidence that exposure to intermittent hypoxia has beneficial effects such as increasing ischemia resistance and preserving cardiovascular and brain function, also described as ischemic preconditioning [6].

Ischemic preconditioning potentially affects postoperative function via numerous pathways, including the regulation of neurotrophin expression, strengthening the neurovascular network decreasing inflammation and apoptosis [7]. However, most studies were based on rodents and the translation to humans remains to be evaluated. On the other hand, the expression of brain derived neurotrophic factor (BDNF) is correlated positively with cognitive function in older adults [8]. Furthermore, intermittent hypoxia stimulates the expression of BDNF in the adult hippocampus [9] and enhances the process of learning [10]. Hence, intermittent hypoxia might result in an improvement in cognitive function. One may therefore hypothesize that the hypoxemic intervals associated with OSAS may have a beneficial effect on the postoperative cognitive function in terms of an ischemic preconditioning. We investigated this question in a pilot study in patients with a high risk of OSAS and in control patients undergoing non-cardiac surgery with total intravenous anesthesia.

## Methods

In this prospective single-center study, two groups (OSAS group and a control group) were compared. The study was performed at the University Hospital of Erlangen, Germany between June 2012 and June 2013 in accordance with the guidelines for Good Clinical Practice and the Declaration of Helsinki. The study was approved by the local Ethics committee (Ethikkommission der Medizinischen Fakultät der Friedrich-Alexander-Universität Erlangen-Nürnberg, Erlangen, Germany) on 19.04.2012 (reference number: 87_12 B).

### Patients

After written informed consent, 51 adult patients of both genders with an estimated hospital stay of at least 3 days undergoing surgery were enrolled in this study. Inclusion criteria were an age between 55 and 80 years and an American Society of Anesthesiologists physical status classification of I to III. Patients with a history of brain or head injury, cerebral ischemia, diseases of central nervous system, psychological disorder, alcohol or illegal drug abuse, neuroenhancing or neurocompromising medication, manifest diagnosis of pre-existing cognitive impairment or severe cardiovascular disorder were excluded from the study. Patients were assessed by a detailed screening examination and clinical interview. None of the included patients had been treated for OSAS. Cognitive function was assessed using a neuropsychological test battery on the day before surgery as a baseline measurement. The postoperative testing was performed after surgery on the first or second postoperative day.

### Assessment of the risk for OSAS

The severity and classification of OSAS is most commonly characterized by measuring the apnea/hypopnea index (AHI) resulting from respiratory disturbances while the sleeping patient is monitored in a sleep laboratory. Oxygen desaturation far below the physiological range is the key clinical parameter to assume an apnea episode during sleep. In the present study, however, we used the STOP-BANG test to identify patients with a high risk for OSAS. The STOP-BANG test is a brief, well-known and international accepted questionnaire for sleep disorders [1]. This validated test detects patients with an increased risk for OSAS [11, 12]. The STOP BANG questionnaire asks for the incidence of snoring, fatigue and observed stop of breathing, and considers also data on blood pressure, body mass index, age, neck size and gender. Patients meeting the study criteria were asked to participate in the study. The assessors were blinded to the screened and enrolled patients. Patients were screened during premedication visit of the anesthesiology staff. Eligible patients were enrolled after written informed consent to the study. After consent, the patients were screened via the STOP Bang Test. If the resulting composite score (range 0 to 8) was 3 or higher, the patient was assigned to the OSAS group, otherwise the patient was assigned to the control group. The study was performed by two assessors to ensure a testing phase within the study schedule. There were no significant differences between both assessors regarding the results validity detected.

### Clinical protocol

Preoperatively, we registered all medications, which had been prescribed and assured that no neuroenhancing or neurocompromising medication had been started recently or was given routinely. Benzodiazepines for premedication were strictly avoided in all patients, and instead 75 or 150 μg clonidine was given orally if needed. Total intravenous anesthesia with propofol and remifentanil was performed in all patients. Prior to intubation, patients received 0.1 mg fentanyl. Propofol and remifentanil were administrated as target controlled infusion (TCI). Rocuronium was given as neuromuscular blocking drug. Intraoperatively hemodynamic stability was adjusted in order to avoid hypotension and hypertension phases or fluctuations of blood pressure while surgery.

Hemoglobin oxygen saturation was monitored in all patients by pulse oximetry. Prior to in- and extubation, all patients received 100% oxygen for several minutes and were transferred quickly to the recovery room. If necessary, additional oxygen was supplied in the recovery room and patients were weaned of oxygen support prior discharge.

During the postoperative phase, control patients were monitored in the recovery room for at least two hours. Patients in the OSAS group were monitored overnight according to our hospital’s clinical security guidelines. Pain was evaluated by the 11-point numerical rating scale (NRS, 0 = no pain, 10 = maximum pain). If the patient’s pain score was greater than 4, an infusion with 7.5 or 15 mg piritramide was administrated over 20 min according to total body weight.

### Assessment of cognitive function

Cognitive function was assessed using a neuropsychological test battery comprising six different cognitive test as a baseline measurement on the day before surgery. The postoperative testing was performed after surgery on the first or second postoperative day. All perioperative tests were carried out in a quiet and separate room during daytime and it was attempted to perform pre- and postoperative tests at matching times of the day. All tests required approximately 60 min per testing phase and patient. To avoid learning effects, tests were presented in two different versions if necessary. The following tests were performed:

### DemTect

The DemTect is a highly sensitive psychometric screening test to identify patients with mild cognitive impairment and patients with dementia in the early stages of the disease [13]. The test consists of five tasks, which survey the functions “verbal memory”, “verbal fluency”, “cognitive flexibility” and “attention”. The transformed total score with a range from 0 to 18 points is independent of age and education. DemTect helps in deciding whether cognitive performance is adequate for age (13–18 pts.) or whether mild cognitive impairment (9–12 pts.) respectively dementia (0–8 pts.) is likely.

### Rivermead Behavioural memory test (RBMT)

The Rivermead Behavioural Memory test is a highly sensitive test of global memory impairment examining immediate and delayed recall [14]. It is designed to predict daily memory difficulties in people with an acquired and non-progressive brain injury in order to monitor their capability in the course of time. Regarding four parallel forms, learning effects can be avoided during the testing phases. We selected the subtest “story” for our study in which a nearly 55 word story is read out to the subject who has to recall the content immediately and after a 25 min interval. This subtest is known as a suitable evaluation of verbal memory function, logical memory and episodic memory. Maximum score is 84 points and a higher test value means a better performance.

### Zahlen-Verbindungs-test (ZVT)

The ZVT measures general intelligence performance and analyzes non-verbal cognitive performance speed independent of education but influenced by genetics [15]. The corresponding ability is quantified as “liquid intelligence” or “perceptual or processing speed”. The test represents a diagnostic tool that is used clinically in organic brain disease. Tested subjects have to connect 90 ascending numbers, arranged randomly on four different sheets of paper with a pen. The time needed is recorded, averaged and age-adjusted for interpretation. Thus, a higher test value means a worse performance.

### Trail-making-test (TMT)

The Trail Making Test A/B attempts to test neurocognitive performance combined with psychomotor ability by using a setting in which the patient has to connect up scaling numbers in the correct order [16]. In the B version the test requires an additional task by switching between alternating numbers and letters. The time needed is recorded, and consequently longer total times reveal greater impairment. In our study we used TMT B, which provides information about visual search speed, scanning, speed of processing and mental flexibility, which are good parameters for executive function.

### Digit span test

The Digit Span Test used in our study is a subtest of the Wechsler Memory Scale [17]. It consists of random number sequences presented orally to the patient. The single digits have to be repeated in the same order. If repeated correctly, a single digit will be added to the sequence. The examination is repeated twice forwards and twice backwards. Each examination uses six numerical series. Test scoring is based on the total digits recalled correctly. Each correct answer leads to one point. Maximum score is 24 and higher scores indicate better cognitive performance. The Digit Span Test investigates the patient’s short term memory capacity (digit span forward) and verbal working memory (digit span backward).

### Short cognitive performance test for assessing deficits of memory and attention (SKT)

The SKT assesses memory and attention deficits within a clinical setting [18]. It consists of nine subtests, which retrieve immediate and delayed verbal memory and attention, measured as the speed of information processing. Test samples are available in five parallel forms to avoid learning effects. Raw scores from each subtest are converted into norm values and a total score, which are age adjusted. Due to its subtest structure, memory and attention can be assessed separately. The scores range from 0 to 9 points for memory, and from 0 to 18 points for attention, respectively. Hence, the total score ranges from 0 to 27 points. Notable is that higher scores indicate more severe cognitive impairment, which is given gradually for each test result and related to the status of impairment.

### Color-word-interference-test (FWIT)

The FWIT [19] is assembled from the three following parts: reading written color-words, naming the ink-color of a printed line and naming the ink-color of a written color-word instead of reading the word itself, which names a different color. In each part, processing time and the number of errors are recorded. Thus, a higher test value means a worse performance. The test measures nomination, alertness, and selectivity or rather interference. Recorded data allow the interpretation of executive function. The advantage of this test is that it is not subject to learning-effects upon repetition.

### Statistical analysis

Outliers were identified using the Grubbs test. In this test, the value Xmax which shows the largest deviation from the population mean is identified as outlier and removed from the data set if the test statistic Z = abs(mean-Xmax)/SD is larger than a critical value which depends on the sample size. In our case with 21 and 22 patients in the two groups, the critical values for *p* < 0.05 were 2.73 and 2.76, respectively. The procedure is iterated until no outliers are further detected. Categorical data were tested for differences between the two groups using the chi-square test. Continuous data were tested for deviations from the normal distribution using the Shapiro-Wilk test. The primary outcome parameter was the change of the cognitive function assessed by the difference between postoperative and preoperative test scores within a subject. The change from baseline values to post-anesthesia values within a group was tested for statistical significance using the paired t-test or the Wilcoxon test, respectively. The change of the cognitive function was further tested for significant differences between the two groups using the unpaired t-test or the Mann-Whitney test, respectively. In order to account for a different distribution of males and females within the two groups, we also analyzed the change of the cognitive function by ANOVA with the factors “gender” and “group”. The level of significance was defined as *p* < 0.01.

The sample size was estimated based on published results for the DemTect [13]. A standard deviation of approximately 3 points was reported for the score. We assumed a difference of at least 4 points between the two groups as clinically relevant. Therefore, we needed at least 19 subjects per group to detect such a difference with an α-error of 0.01 and a power of 0.9. As we expected a 25% dropout rate for the postoperative testing we aimed a study size of at least 25 patients per group.

Categorical data are reported as numbers, continuous data are reported as median and range if not stated else. Statistical analysis was performed using Statistica software (Statistica Version 6, Tulsa/USA).

## Results

### Demographics and clinical data

We recruited a total of 51 patients, out of which seven patients did not complete the second test phase. Reasons for study dropouts were pain, discomfort and inconveniences leading to time shifts in the second phase test protocol. From the remaining patients, one patient was identified as outlier. Demographic and related clinical data of finally 43 patients used for analysis are given in Table 1. The twenty-two patients assigned to the OSAS group had a median score of 5 (3–7) points in the STOP BANG test and therefore a high risk for OSAS, compared with the patients in the control group with a median score of 2 (1–2) points. OSAS patients and control patients did not differ in age, but the percentage of males was significantly higher in the OSAS group. However, this distribution reflects the epidemiology of sleep apnea, which is far more frequent in males. Correspondingly, the patients in the OSAS group had a significantly higher weight and height. As expected, we also found a significant difference in BMI, since obesity is an important risk factor for sleep apnea and both parameters are used in the STOP BANG questionnaire.

**Table 1:** Patients‘data

	Control (*n* = 21)	OSAS (*n* = 22)	*P* value
Demographics			
Age (yrs.)	64 (57–72)	66 (60–76)	0.50
Gender (female/male)	13/8	4/18	0.004
Body weight (kg)	74 (61–100)	90 (74–112)	0.0002
Height (cm)	168 (155–183)	177 (159–188)	0.006
Body mass index (kg m^− 2^)	27 (22–35)	29 (23–38)	0.037
ASA classification (I/II/III)	2/16/3	0/16/6	0.23
STOP-BANG score	2 (1–2)	5 (3–7)	
Comorbidities			
History of smoking	6	10	0.48
Diabetes mellitus	1	4	0.16
Surgery			
Otorhinolaryngologic surgery	13	12	
Other type of Surgery	8	10	
Operation data			
Duration of operation (min)	70 (25–186)	76 (17–195)	0.61
Duration of anaesthesia (min)	113 (39–330)	140 (42–236)	0.38
SpO_2_ before procedure (%)	97 (92–100)	96 (92–100)	0.059
SpO_2_ at extubation (%)	99 (91–100)	100 (81–100)	0.97
SpO_2_ at arrival intermediate care (%)	96 (88–100)	93 (88–98)	0.080
SpO_2_ lowest level (%)	91 (70–98)	92 (72–99)	0.72
Propofol dose (mg kg^− 1^ h^− 1^)	6.18 (5.45–11.7)	5.84 (0.84–7.03)	0.11
Remifentanil dose (μg kg^− 1^ min^− 1^)	0.16 (0.11–0.33)	0.17 (0.09–0.23)	0.64

Data are reported as number or as median (range). SpO_2_ is the oxygen saturation measured by pulse oximetry. *P* value is the significance level of the difference between the two groups

The main type of surgery was otorhinolaryngologic surgery. There were no significant differences regarding dosage and anesthesia procedure between the OSAS and the control group. There were also no differences in oximetry parameters in both groups, although it’s notable that we found a slightly more pronounced desaturation in the OSAS subjects after transfer to the recovery room, when the mean oxygen saturation was 96% in the control group and 93% in the OSAS group, respectively.

### Neuropsychological testing

Cognitive function was assessed using a combination of neuropsychological tests on the day before surgery as a baseline measurement. The same test battery was repeated on the first or second postoperative day. Thirty five patients out of 43 patients were tested on the first postoperative day and only eight (three control and five OSAS patients) on the second postoperative day. The pre- and postoperative test results are summarized in Table 2. Preoperatively, significant differences between the two groups were found only for the DemTect, where the patients in the OSAS group showed a significant worse performance compared to the control group (*p* = 0.0043). When comparing pre- and postoperative values within the control group, there was a significant decrease in three of the tests (DemTect, RMBT, and SKT). In contrast, the patients in the OSAS group did not show a significant loss in any of the tests but even a significantly improved performance in the RMBT test. The differences between the two groups were more obvious when not the absolute values but the difference of the post- and the preoperative values were compared (Table 3). In five of the eight tests (DemTect, RMBT, Digit span forward and backward, and SKT), we found a significant difference between the two groups with respect to the change from pre- to postoperative cognitive function. This statistical significance was confirmed by the ANOVA which revealed high significance for the factor “group” but no significance for the factor “gender” and the interaction between the two factors.

**Table 2:** Results of the pre- and postoperative neurocognitive testing

Test	Control group			OSAS group		
	Pre	Post	*P* value	Pre	post	*P* value
DemTect (points)	18 (13–18)	14 (8–18)	< 0.0005	16 (12–18)	15 (12–18)	0.81
RBMT (points)	39 (14–58)	25 (6–47)	0.0024	35 (16–51)	37 (15–58)	0.0041
ZVT (mm:ss)	02:00 (01:13–03:32)	01:47 (00:55–2:51)	0.019	01:47 (01:06–2:59)	01:43 (01:03–2:49)	0.0055
TMT (mm:ss)	01:59 (00:47–03:49)	02:14 (00:53–3:20)	0.11	01:43 (00:42–04:30)	01:32 (00:34–3:34)	0.22
Digit Span Forwards (points)	9 (6–12)	7 (4–12)	0.010	8 (5–11)	9 (4–12)	0.033
Digit Span Backwards (points)	7 (3–10)	5 (3–10)	0.011	6 (4–11)	8 (3–11)	0.050
SKT (points)	1 (0–7)	5 (1–9)	< 0.0005	3 (0–8)	3 (1–7)	0.24
FWIT (mm:ss)	00:56 (00:40–01:19)	00:58 (00:43–1:16)	0.31	00:57 (00:45–01:15)	00:55 (00:48–1:24)	0.09

Data are reported as median (range). The *p* value is the significance level of the difference between pre- and postoperative values within a group

**Table 3:** Change of cognitive functions, expressed as the difference between post- and preoperative values

Test	Control	OSAS	P_MWU_	P_ANOVA_
DemTect (points)	−4 (−8–0)	0 (− 4–6)	< 0.0001	< 0.0001, 0.56
RBMT (points)	−15 (− 25–25)	5 (−9–16)	< 0.0001	< 0.0001, 0.91
ZVT (min)	− 0.17 (− 1.55–0.29)	−0.16 (− 0.63–0.29)	1.00	0.26, 0.32
TMT (min)	0.19 (−1.44–0.79)	- 0.11 (− 1.67–1.16)	0.049	0.37, 0.35
Digit Span Forwards (points)	−1 (− 6–2)	1 (− 2–3)	0.0013	0.0010, 0.74
Digit Span Backwards (points)	−1 (− 4–3)	2 (−3–5)	0.0025	0.0019, 0.91
SKT (points)	3 (−3–6)	1 (− 6–5)	0.0022	0.026, 0.31
FWIT (min)	0.03 (−0.18–0.26)	−0.02 (− 0.23–0.33)	0.055	0.28, 0.59

Data are reported as median (range). P_MWU_ is the significance level of the difference between the two groups obtained by the Mann-Whitney test. P_ANOVA_ are the significance levels of the factors “group” and “gender” obtained by analysis of variance

## Discussion

It was the aim of this study to evaluate the impact of a high risk of OSAS on the postoperative cognitive dysfunction after intravenous anesthesia. Patients susceptible for a high risk of OSAS were identified using the STOP BANG questionnaire, which is recommended for screening patients with moderate to severe risk for OSAS [20–22]. Recently, Kim and co-workers published a very high sensitivity of 97% for the STOP BANG questionnaire [23]. However, its specificity was low (19%). More important for our purpose was the finding that the positive predictive value was quite high with 86%. Thus, this test seems to be able to detect patients with an at least moderate risk for OSAS. Moreover, several other recently published validation studies revealed fairly good positive predictive values to identify patients with sleep breathing disorder [24–26]. Furthermore, in a systematic review prepared as a part of the Society of Anaesthesia and Sleep Medicine (SASM) publication for the Preoperative Assessment of Patients with Sleep Disordered-Breathing, Opperer and co-workers analyzed how the diagnosis OSA was elucidated. Based on the SASM consensus group criteria for OSAS, 17 publications referred to the diagnosis via polysomnographic results and 15 published articles on screening questionnaires [27]. Chung and co-workers reported that a STOP-Bang score of 5–8 identified patients with a high probability of moderate or severe OSAS in a surgical population [22]. Therefore, we assumed that the patients in our OSAS group were highly suspected of having intermittent hypoxemic episodes.

To account for possible interindividual variation with respect to education levels, we focused on the change in test results prior to and after surgery between OSAS and control patients. In most tests performed, we found significant differences in the change of memory function between OSAS patients and control group patients. OSAS patients in general showed a significantly less decrease or even an increase of the performance compared to the control group.

In this study six separate cognitive test were used to detect different aspects of cognitive impairment as described above. While some tests address a single cognitive function, like the Digit Span test in terms of memory or the RMBT in terms of several aspects of memory, other tests address multiple aspects of cognitive function. Gagnon and colleagues summarized that OSAS patients elicited in all cognitive functions an impaired ability compared to controls [28]. Focusing on the attention aspect, the DemTect and SKT in our study elicited a decrease in attention performance. Moreover, executive function was tested with both tests, DemTect and SKT. Executive function is a complex system of skills including behavioral inhibition, mental flexibility, and working memory. We found impairments in executive function in the preoperative testing phase in OSAS patients compared to controls as described by others [28]. Focusing on executive function, which was tested separately by the FWIT, the OSAS group presented diminished results in our study preoperatively. Working memory was tested separately by the Digit Span test, which likewise showed impaired results in the OSAS group preoperatively. Psychomotor speed was analyzed via TMT und ZVT which revealed decreased results in OSAS patients. Memory function was evaluated using the RMBT, in which the OSAS subjects elicited decreased results in comparison to controls. These results of the present study are in accordance with previously reported findings [28].

Postoperatively, the DemTect revealed a decrease in verbal memory performance in control patients but not in OSAS patients. Using the RBMT, verbal function was also found to be impaired significantly in control patients, whereas it even improved significantly in the OSAS group. Working memory and memory capacity as assessed by the Digit Span Test were also characterized by an impairment in controls but an improvement in OSAS patients. A similar aspect of memory function is the quality of attention capability, as rated by the SKT test. Again, the test performance of the OSAS group improved after surgery, whereas the performance of the control patients clearly deteriorated. On the other hand, the Color Word Interference Test, which investigates the ability of nomination, alertness and selectivity or interference respectively executive function, did not reveal significant differences between both groups. Moreover, the ZVT did not detect any differences between both groups representing liquid intelligence or processing speed. Finally, using the TMT, which aimed at detecting processing speed as well as rating executive function, we found only minor differences between both groups’ test performance.

It is well believed that OSAS patients have impaired cognitive function [29]. There is, however, controversy regarding the grade of impairment. As there is a wide variety in neurocognitive test systems it is difficult to compare the results [30]. The patients in our OSAS group showed indeed a worse preoperative performance in the Demtect test. However, these patients stayed at this level whereas the patients in the control group showed a significantly reduced test performance postoperatively than reported in the literature [31]. Regarding memory function ability, we used the Rivermead Behavioural Memory Test to focus on logical and episodic memory function. In the literature, memory function has been assessed differently and different aspects of memory function are emphasized. While some reviews found impairments in short-term memory function [32], others reported deficits in verbal and visual delayed long-term memory in OSAS patients [33]. On the other side, no effect was found analyzing the severity of OSAS and its influence on memory function [34]. We found that individuals with a high risk of OSAS performed significantly better in a combination of logical and episodic test settings than control subjects. We concentrated on verbal memory using the subtest “story” in its immediate and delayed version. While we found an increase in verbal memory performance between both test intervals, others described a decrease regarding verbal episodic memory tasks [35].

The observation that the OSAS patients in our study generally showed a less postoperative decrease or even an increase of the cognitive function might be explained by the phenomenon of hypoxic preconditioning. It is well known that OSAS patients show nocturnal oxygen desaturation [2]. Affected patients present significant consequences including excessive daytime sleepiness. Experimental studies in healthy adults with sleep deprivation lead to the assumption that sleepiness negatively influences neurocognitive performance [36, 37]. Others reported that sleep deprivation tolerance in young adults might compensate alteration of cognitive capabilities [38]. Further investigations demonstrated the existence of an ample cognitive reserve and cerebral recruitment enabling individuals to maintain cognitive performance and to resist sleep deprivation induced impairment [39, 40]. Functional imaging studies confirmed this protective mechanism in OSAS patients [41]. This observation might explain the finding that patients affected by mild or moderate OSAS can present a normal cognitive function [42]. On the other hand, the severity of hypoxemia altered processing and motor speed performance [42]. It is well known that severe unrecognized and unanticipated desaturation phases may occur following general anesthesia [5]. In both study groups we detected oxygen desaturation after the surgical intervention (Table 1). While our postoperative observations mostly revealed a single hypoxic stimulus following anesthesia randomly, OSAS patients are exposed to multiple hypoxic stimuli per night. Given the assumption that OSAS patients might have adapted to those numerous desaturation events, a single, fortuitous ischemic strike might not negatively affect the neurocognitive performance in these patients. Beneficial effects of prior episodes of ischemia on brain function were found in patients with transient ischemic attack [43]. Hoth and co-workers reported that subjects with a more pronounced hypoxemia performed better in learning and memory tasks compared to those with less severe levels of hypoxemia [44]. These findings led to the assumption that ischemic preconditioning might have protective effects on brain function. Consequently, remote preconditioning approaches have been proposed [45, 46]. However, a recently published study failed to prove beneficial effects of remote ischemic preconditioning on postoperative neurocognitive dysfunction in patients who underwent cardiac surgery [47]. On the other hand, lately published data suggest that remote ischemic preconditioning prevent a short-term postoperative cognitive function decline after cardiac surgery [48]. Moreover, He and co-workers published recently that remote ischemic preconditioning improved cognitive function in elderly patients [49]. Already in 2013, Schega and colleagues assumed that additional intermittent hypoxic training and physical exercise enhance cognitive function and quality of life in elderly [50]. Recently in a review published by Li and co-workers, it was summarized that ischemic preconditioning techniques could prevent organ damage and play a neuroprotective role [51]. Therefore, we assume that endogenous hypoxic training might be a favorable cerebral preconditioning factor in OSAS patients and leads to clinical relevance in future.

However, there are some study limitations that should be paid attention to. We merely screened the risk for an OSAS which leads to apnea phases while sleep. Patients have not been diagnosed for OSAS using a sleep laboratory tests or polysomnographic studies. However, the STOP BANG test is an international well established screening tool to quantify the risk for having the OSAS. Although patients were comparable regarding surgical procedures, surgery duration time and severity, they were different in weight and body mass index due to the risk factors for OSAS. The patients of the two groups were of similar age, but the gender distributions were different with a significant higher proportion of males in the OSAS group. However, a two-way ANOVA with gender and group as factors revealed that the observed differences in the change of the cognitive function could be attributed to the factor group whereas the gender did not show a significant effect. Moreover, the study focused on non-cardiac surgery patients only. As O’Brien and colleges summarized that intraoperative hypotension is an intrinsic risk factor of postoperative cognitive dysfunction [52], intraoperatively hemodynamic stability was considered conscientiously in this study. We did not observe hypotension, defined as systolic blood pressure values below 80 mmHg [3]. However, brief hypotension phases between the measurement intervals might remain undetected and even small periods of hypotension are clearly associated with worse cognitive function in the postoperative phase. Furthermore it is notable that the heterogeneous results reported in literature could be explained by the various severity of OSAS and the wide range in subjects’ intellectual function and education. Lastly, this study included only total intravenous anesthesia to avoid an additional confounding factor. However, volatile anesthesia should also be tested and analyzed as cognitive impairment is possible in both general anesthesia regimens.

## Conclusions

In conclusion, this is the first prospective study that elicited indications for beneficial effects of possible intrinsic hypoxic preconditioning in patients suspected of being at high risk for OSAS on the early postoperative cognitive function. Focusing on memory function and work memory performance after anesthesia for non-cardiac surgery, we discovered significant differences between patients suspect to sleep apnea and control patients. In a clinical setting, the DemTect or SKT can be recommended to assess cognitive impairment in a fast and valid manner. Cognitive reserve and learning ability might play a role in compensating deficits in executive function. Further investigations are needed to address the impact of the anesthesia regimen.

## Abbreviations

AHI: Apnoea/Hypopnea Index
ASA: American Society of Anesthesiologists physical status classification
BDNF: Brain Derived Neurotrophic Factor
FWIT: Color-Word-Interference-Test
NRS: Numerical Rating Scale
OSAS: Obstructive Sleep Apnea Syndrome
RBMT: Rivermead Behavioural Memory Test
SASM: Society of Anaesthesia and Sleep Medicine
SKT: Short Cognitive Performance Test
STOP-BANG: Snoring, Tired, Observed, Pressure, Body mass index, Age, Neck size, Gender
TCI: Target Controlled Infusion
TMT: Trail-Making-Test
ZVT: Zahlen-Verbindungs-Test
